# Cohort Profile Update: The 1982 Pelotas (Brazil) Birth Cohort Study

**DOI:** 10.1093/ije/dyv017

**Published:** 2015-03-02

**Authors:** Bernardo Lessa Horta, Denise P Gigante, Helen Gonçalves, JanainaVieira dos Santos Motta, Christian Loret de Mola, Isabel O Oliveira, Fernando C Barros, Cesar G Victora

**Affiliations:** ^1^Postgraduate Program in Epidemiology, Universidade Federal de Pelotas, Pelotas, Brazil and ^2^Postgraduate Program in Health and Behavior, Universidade Católica de Pelotas, Pelotas, Brazil

## Abstract

In this manuscript, we update the profile of the 1982 Pelotas Birth Cohort Study.In 1982, 5914 live births whose families lived in the urban are of Pelotas were enrolled in the cohort. In 2012–13, we tried to locate the whole original cohort; 3701 participants were interviewed who, added to the 325 known deaths, represented a follow-up rate of 68.1%. In contrast to the previous home interviews, in this wave all participants were invited to visit the research clinic to be interviewed and examined. The visit was carried out at a mean age of 30.2 years and mainly focused on four categories of outcomes: (i) mental health; (ii) body composition; (iii) precursors of complex chronic diseases; and (iv) human capital. Requests for collaboration by outside researchers are welcome.

**Key Messages**

- It is possible to recruit a population-based cohort and achieve high follow-up rates after 30 years in a middle-income setting.
- The existence of three other younger birth cohorts in the same population allows the evaluation of time trends in health indicators.
- In a cohort where undernutrition was common in early life, extremely high prevalence of overweight and obesity are observed at the age of 30 years.

## What is the rationale for the new focus?

In our previous cohort profile,^1^ we described how all births that occurred in1982 in Pelotas, a southern Brazilian city, were identified. Liveborns whose families lived in the urban area of the city were followedup until adulthood. The 1982 Pelotas birth cohort is considered one of the largest and longest-running birth cohorts in low- and middle-income countries.^2^ The early phases of the study have provided valuable data on the consequences of infant feeding for child health^3^^,^^4^ and on risk factors for infant mortality and undernutrition.^5^^,^^6^ Over time the focus of the study changed, and recent visits evaluated the frequency of precursors of chronic diseases and their risk factors. We have assessed the long-term consequences of early exposures, such as caesarean sections,^7^^,^^8^ infant feeding patterns^9^ and early growth.^10^^,^^11^

Non-communicable diseases (particularly cardiovascular disease, cancer, asthma, chronic obstructive pulmonary disease (COPD), obesity, type 2 diabetes and depression) are the major contributors to the burden of disease, in high-income as well as in middle-income countries.^12^ Although the causes of these epidemics are not fully understood, exposures taking place *in utero* and in early life are consistently associated with their occurrence.^13^^,^^14^ Models of chronic complex disease aetiology tend to focus on risk factors and physiological states relatively close (proximal) in time to the onset of disease, giving limited if any emphasis to processes that lead up to the peak or optimal phenotypic state that is achieved in late adolescence or early adulthood. Studies of the peak phenotypic state are important given its major potential role in influencing susceptibility to complex chronic diseases, being a key component of the developmental models of disease causation.^15^

The project received a grant from the Wellcome Trust, which enabled us to equip our new university headquarters with state-of-the-art equipment for the measurement of body composition, physical activity, lung function and other precursors of chronic disease, allowing the assessment of these phenotypes in young adults.

## What will be the new areas of research?

From June 2012 to February 2013, all cohort members were invited to visit the research clinic in order to be interviewed and examined. The main outcomes in the 30-year follow-up included precursors of complex chronic diseases and their risk factors such as body composition, physical activity and diet. Furthermore, human capital and mental health outcomes were also evaluated. By using data gathered in the previous wave of the cohort, we plan to evaluate the effect of socioeconomic trajectories, early-life exposures and gene-environment interactions on the main outcomes.

## Who is in the cohort?

Previous to the 30-years visit, follow-up visits were carried at the mean ages of 1, 2, 4, 13, 15, 18, 19 and 23 years. Most visits included subsamples of the cohort, except for those at 2, 4 and 23 years. In the last, we attempted to locate the whole cohort. Initially, a city census was carried out in search of cohort members, as many of them had changed address since the previous round. Those who were not located during the census were sought at their most recent available address. Participants answered a questionnaire and were examined at home, and then invited to visit the research laboratory to donate a blood sample, collected by venipuncture.

In February 2012, the research team tried to trace cohort members and update information on addresses and phone numbers. We tried to locate all participants who were not known to have died, using multiple strategies. Initially, they were sought at their last known address; those who were not located were sought at existing databases (university databases, telephone directories and social media). This strategy allowed us to locate 4534 members. A total of 3701 were interviewed, 467 were living far from Pelotas, 86 refused and another 280—although not having openly refused—did not attend the clinic in spite of repeated invitations.The follow-up rate was estimated by adding the number of interviews (*n* = 3701) to the number of participants known to have died (*n* = 325); these made up 68.1% of the original cohort. The mean age at interview was 30.2 years.

Table 1 shows that follow-up rates in 2012–13 were slightly higher among females, those who were born preterm and those in the intermediate socioeconomic categories, whereas birthweight was not related to attrition.

**Table 1.** Follow-up rate at 30 years of age according to baseline characteristics of the cohort

Variable	Original cohort (number)	Followed at 30 years^a^
Sex		
Male	3037	65.2%
Female	2876	71.1%
Birthweight (g)		
< 2500	534	72.1%
2500–2999	1393	69.1%
3000–3499	2220	66.0%
≥ 3500	1762	68.6%
Gestational age (weeks)		
37	294	74.5%
≥ 37	4380	68.1%
Family income at birth (minimum wages)		
< 1	1288	66.1%
1.1–3	2789	70.4%
3.1–6	1091	69.3%
6.1–10	382	61.3%
≥ 10	335	60.3%
Maternal schooling (years)		
0–4	1960	68.0%
5–8	2454	70.5%
9–11	654	66.1%
≥ 12	839	62.8%

^a^Those participants who were known to have died were considered as followed.

## What has been measured?

Unlike the previous visits, this wave of the cohort was carried out at our research clinic. Each participant stayed in the clinic for 3–4 h. The visit included an interview, physical examination, collection of biological samples and assessment of physical activity.

### 

#### Interviews

The interview included three sections: confidential, interviewer-applied and computerized food frequency questionnaire. The latter evaluated the participants’ annual intake of 88 food items. Table 2 shows the main categories of the variables collected using questionnaires; mental health and intelligence quotient were evaluated for the first time.

**Table 2.** Main variables collected in the 30 years follow-up of the study

Instrument	Variables
Interviewer-applied questionnaire	Family structure Schooling performance Employment/salary Friendship patterns Religious practices Cigarette smoking Nicotine dependence Alcohol intake Physical activity Community participation and sense of belonging Romantic/intimate relationships: patterns, preferences and history Use of contraceptive methods Recent negative and positive events of household members Morbidity history Use of health services and medicines Offspring (date of birth, birthweight, breastfeeding duration) Body image Bone fracture Violence and accidents Subjective Happiness Scale Use of a food supplement, binge eating and other dietary habits Use of SRQ-20
Self-reported questionnaire	Food frequency questionnaire
Confidential questionnaire	Illicit drug use Frequency of intercourse Number of partners Contraceptive practices Condom use Number of abortions
Psychological interview	Intelligence quotient MINI Major depressive episode Suicidality Manic/hypomanic episode Agoraphobia Social phobia Generalized anxiety disorder Attention deficit / hyperactivity disorder Beck Depression Inventory (BDI-II)

SRQ-20, Self-reporting Questionnaire.

Four psychologists assessed mental health and intelligence quotient. The Mini International Neuropsychiatry Interview V5.0^16^ (MINI) was used to detect the presence of depression, attention deficit / hyperactivity disorder, suicidal ideation, generalized anxiety disorder, agoraphobia and social phobia. The Beck Depression Inventory (BDI-II) assessed the intensity and frequency of depression symptoms. Intelligence was measured using the Wechsler Adult Intelligence Scale, Third Version (WAIS-III).

#### Physical examinations

In the previous visits, the physical examination had consisted of measurements of blood pressure, weight, height and waist circumference. In this wave, an increased number of physical assessments were performed. Body composition was evaluated using dual-energy Xray absorptiometry (DXA Lunar Prodigy) and air-displacement plethysmography (BodPod). A photonic scanner (3-DPS) was used to capture body surface topography, from which extensive body shape information can be extracted using computer algorithms.We relied on traditional anthropometry to measure height, sitting height and waist circumference. The thickness of the adductor pollicis muscle and skinfolds (triceps and subscapular) was assessed with a Lange caliper. Ultrasound examination (Toshiba Xario) was used to measure visceral and subcutaneous abdominal fat, as well as carotid intima-media thickness. Pulse wave velocity was also assessed [Sphygmocor (Atcor Medical, V9.0)]. Spirometry was undertaken using a portable, Easy-One spirometer (Medical Technologies, Zurich, Switzerland) following American Thoracic Society recommended procedures,^17^ before and 15 min after inhalation of 200 µg salbutamol. A saliva sample was collected from a subsample of participants for deuterium body composition analysis (*n* = 204).

For the first time in the cohort, physical activity was evaluated using a GENEActiv accelerometer (ActivInsights, Kimbolton, UK). The monitor was worn on the non-dominant wrist for 4–7 days, and collected at the participant’s home at the end of this period.

#### Blood samples

Blood samples were collected and DNA was extracted from venous blood. Serum, whole blood and DNA samples were stored at adequate temperatures. Total cholesterol, HDL-cholesterol, LDL-cholesterol, C-reactive protein and glycated haemoglobin were measured. In 2012, DNA samples collected in the 2004–05 visits were genotyped using the Illumina Omni 2.5 M array.

## What has it found? Key findings and publications

Data analyses are currently under way, and Table 3 presents some illustrative results that have not been published elsewhere.

**Table 3.** Characteristics of the studied population at the 2004–05 and 2012–13 visits according to mental health, body composition, precursors of complex chronic diseases and human capital variables

Indicator	2004–05		2012–13	
	Men	Women	Men	Women
Mental health				
Prevalence of common mental disorders	23.5%	32.8%	20.0%	31.5%
Prevalence of major depressive episode	NA	NA	4.6%	12.5%
Prevalence of attention deficit / hyperactivity disorder	NA	NA	3.0%	4.2%
Body composition				
Body mass index (kg/m^2^)^a^	23.8 (4.1)	23.4 (4.6)	27.0 (5.0)	26.7 (6.0)
Prevalence of overweight (body mass index > 25 kg/m^2^)	30.7%	25.6%	62.9%	52.4%
Prevalence of obesity (body mass index > 30 kg/m^2^)	7.5%	9.1%	22.1%	23.8%
Visceral abdominal fat thickness (cm)^a^	NA	NA	6.9 (2.0)	4.9 (1.7)
Subcutaneous abdominal fat thickness (cm)^a^	NA	NA	1.9 (1.0)	2.6 (1.2)
Fat mass percentage (BodPod)^a^	NA	NA	24.5 (9.2)	37.4 (8.5)
Precursors of chronic disease				
Systolic blood pressure (mmHg)^a^	123 (14)	111 (13)	128 (12)	115 (12)
Diastolic blood pressure (mmHg)^a^	76 (12)	71 (11)	77 (9)	74 (9)
Carotid intima-media thickness (μm)^a^	NA	NA	585 (21)	579 (15)
Prevalence of tobacco smoking	27.6	23.6	26.0	21.3
Forced expiratory volume in 1 s (l/s)^a^	NA	NA	4.0 (0.6)	2.9 (0.5)
Human capital				
Achieved schooling (years)^a^	9.0 (3.1)	9.8 (3.1)	10.9 (4.0)	11.7 (4.3)
Intelligence quotient (points)^a^	NA	NA	98.5 (12.8)	97.5 (12.4)

NA, data not available.

^a^Mean and standard deviation.

### 

#### 

##### Mental health

The proportion of participants diagnosed as presenting common mental disorders was slightly lower at 30 than at 23 years of age. In both waves, females had higher prevalence than males. In the 2012–13 visit, major depression was also more commonly diagnosed among females than males.

##### Body composition

Over a time span of 7 years, we observed a sharp increase in the mean body mass index, as well as in the prevalence of overweight and obesity. Percent fat mass and subcutaneous fat thickness (assessed through ultrasound) were higher among women, whereas visceral abdominal fat thickness was higher among men.

##### Precursors of complex chronic diseases

Males presented higher blood pressure and carotid intima thickness. Table 3 shows that mean systolic blood pressure increased from 2004–05 to 2012–13. On the other hand, the prevalence of smoking slightly decreased from 23 to 30 years of age, for both sexes.

##### Human capital

Achieved schooling was higher among females, but males participants scored higher in intelligence tests.

## What are the main strengths and weaknesses?

In the 2012–13 visit, we managed to locate 68.1% of the cohort. This follow-up rate is similar to that observed in cohorts from high-income countries at a similar age,^18^^,^^19^ and higher than in other cohorts from LMICs.^20^ Furthermore, we were able to carry out a very thorough physical examination of participants, collecting data on several precursors of chronic diseases and evaluating some phenotypes close to their peak. In particular, this is one of the largest prospective cohort samples with data derived from DXA, plethysmography, abdominal ultrasound and 3-D photonic scanner. This plethora of information will allow a better understanding of the role of early exposures on the programming of chronic diseases and nutritional status.

Another strength is that, for some early exposures (such as breastfeeding), the confounding pattern in terms of early exposures differs from that observed in high-income settings. Comparison of cohorts with different confounding structures may allow more valid assessments of causality.^21^

The 1982 birth cohort included > 99% of all births that took place in the city in that year, and therefore may be regarded as a representative sample of the city’s population. Likewise, we recruited a similar cohort in 1993 and in 2004, and recruitment for the 2015 cohort is currently under way. The existence of four cohorts, 11 years apart, with similar methodology and in the same population, is an important strength of our set of studies.

On the other hand, in spite of being able to follow a high proportion of the cohort, the attrition rate was slightly higher among the very poor and the wealthy participants. Nevertheless, follow-up rates among different subgroups are reasonably similar (ranging from 60% to 75% in all variables studied), so that attrition bias is unlikely.

## Can I get hold of the data? Where can I find out more?

We welcome requests for joint analyses with other cohorts and collaboration with outside researchers, as well as exchange of doctoral or post-doctoral fellows with other institutions. We encourage outside researchers to spend some time in Pelotas to get to know the cohort and the datasets. With respect to collaboration with other cohort studies, our group launched the COHORTS consortium,^20^ and have collaborated with the Avon Longitudinal Study of Parents and Children (ALSPAC).^22^^,^^23^ Collaborations with genetic epidemiology consortia are being established now that our genome-wide association study (GWAS) results are available.

The questionnaires and interviewer guides from all follow-up visits are available in electronic formats at [http://www.epidemio-ufpel.org.br/site/content/coorte_1982/questionarios.php]. Applications to use the data should be made by contacting the researchers of the 1982 cohort and completing the application form for the Pelotas Birth Cohorts available at [http://www.epidemio-ufpel.org.br/site/content/estudos/formularios.php].

## Funding

From 2004 to 2013, the Wellcome Trust supported the 1982 birth cohort study. The Brazilian National Research Council (CNPq) and Rio Grande do Sul State Research Support Foundation (FAPERGS) also supported this phase of the study. The International Development Research Center, World Health Organization, Overseas Development Administration, European Union, National Support Program for Centers of Excellence (PRONEX) and the Brazilian Ministry of Health supported previous phases of the study.
